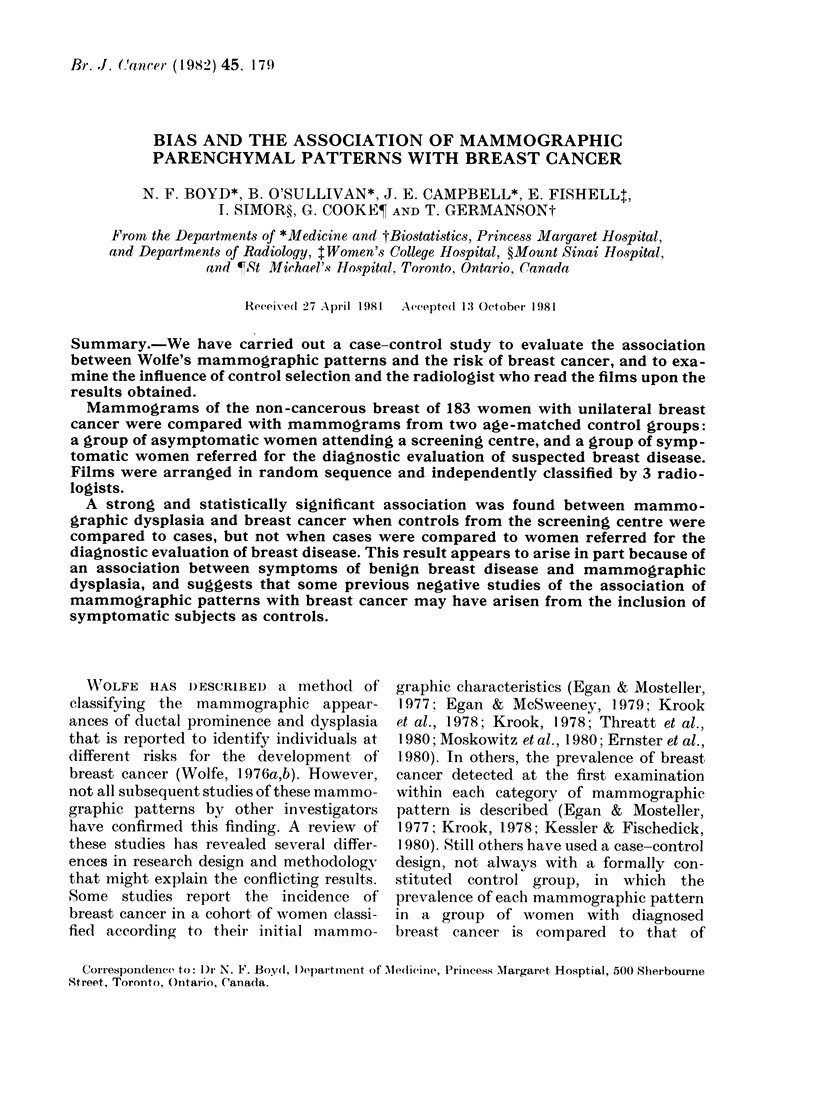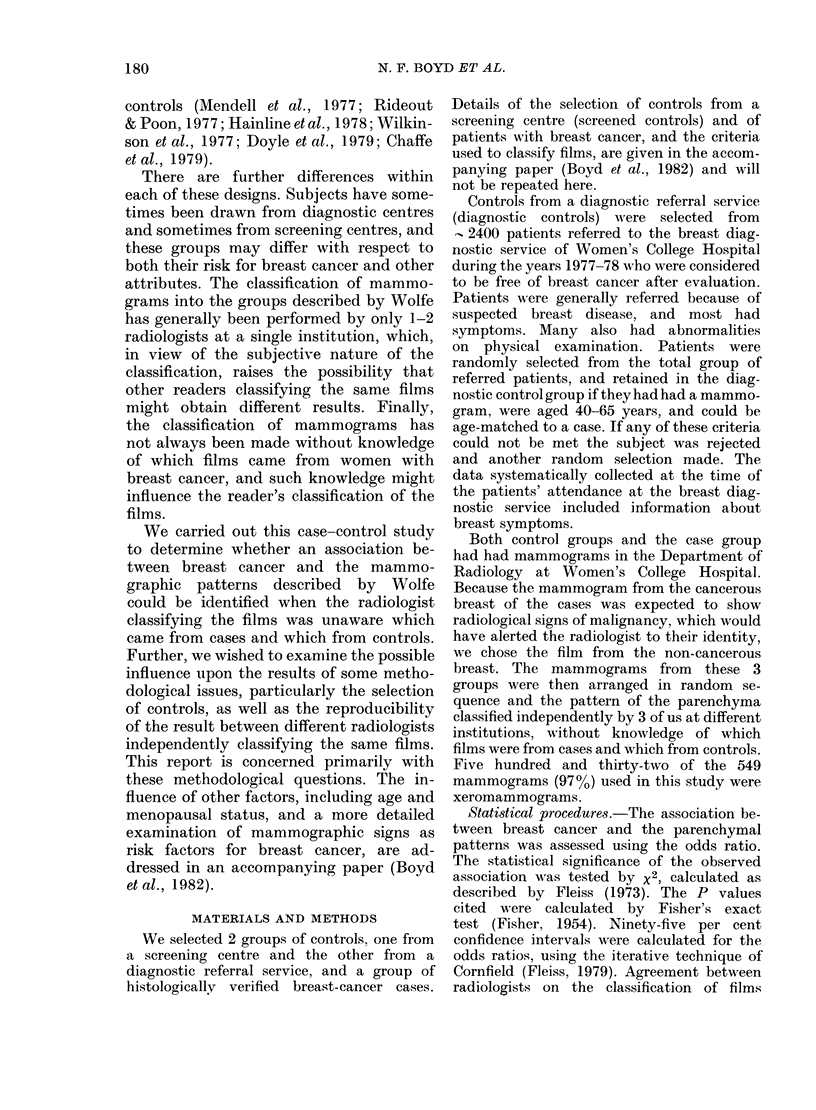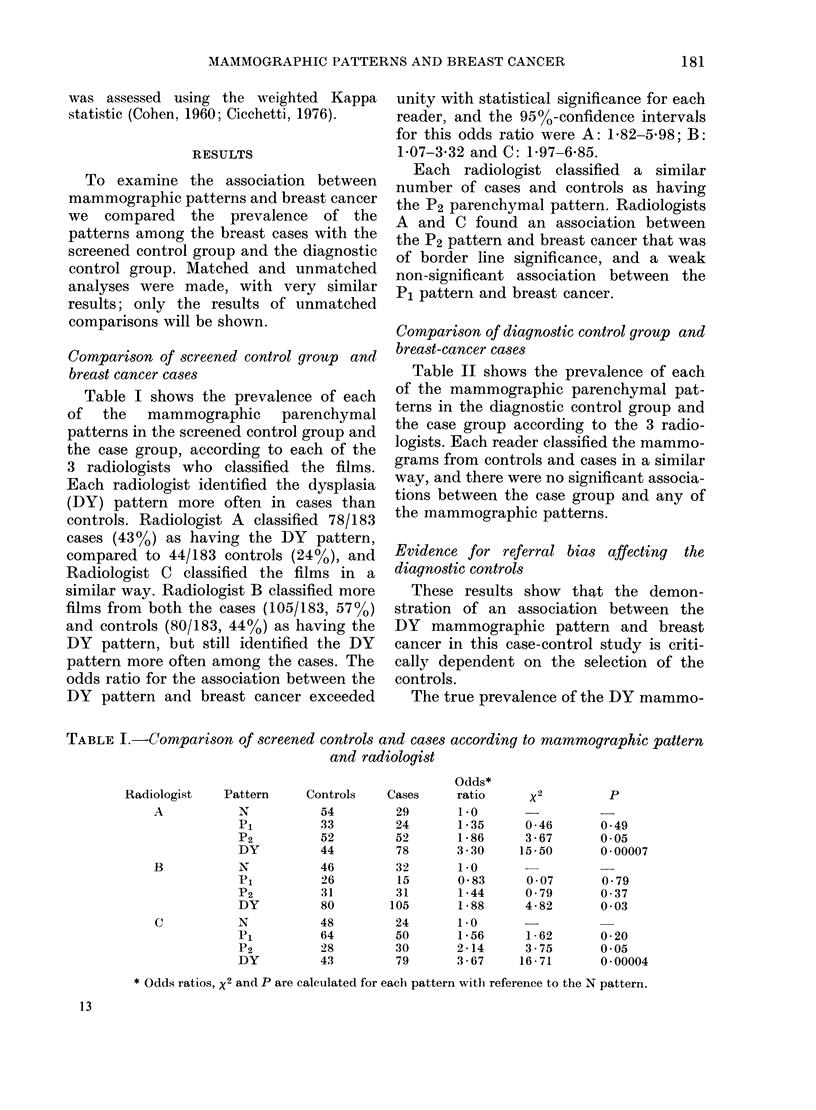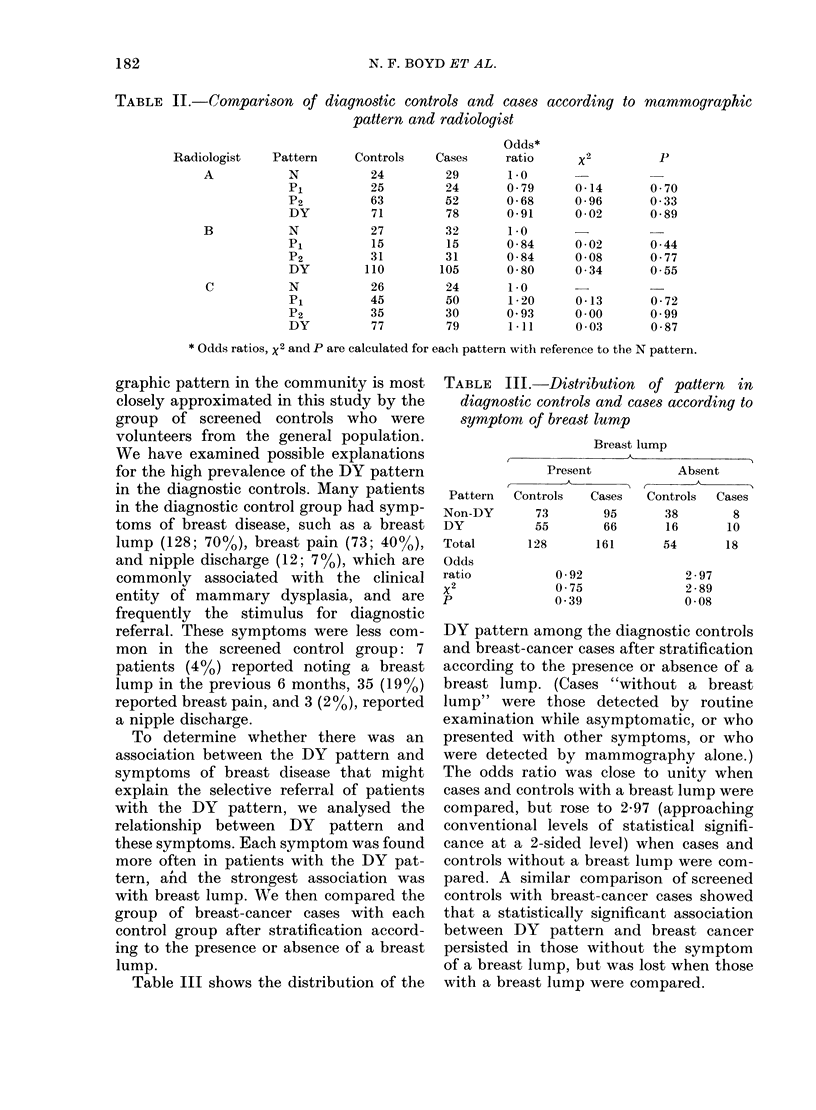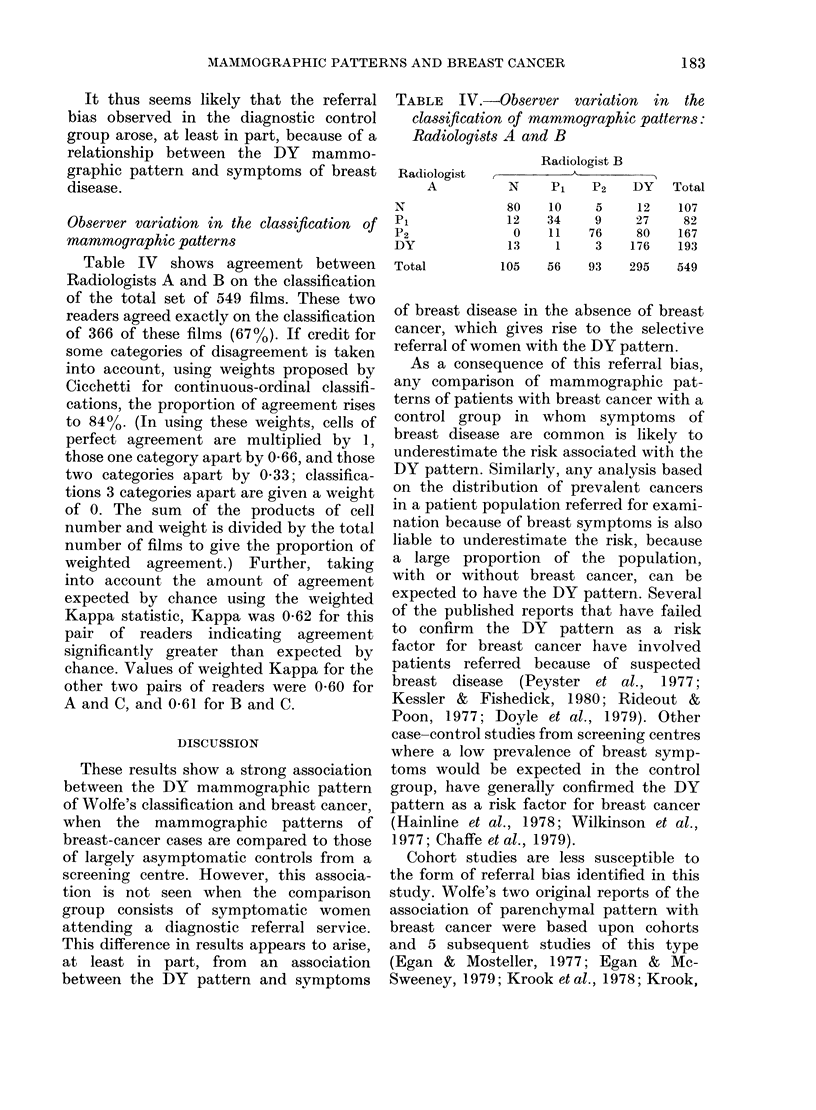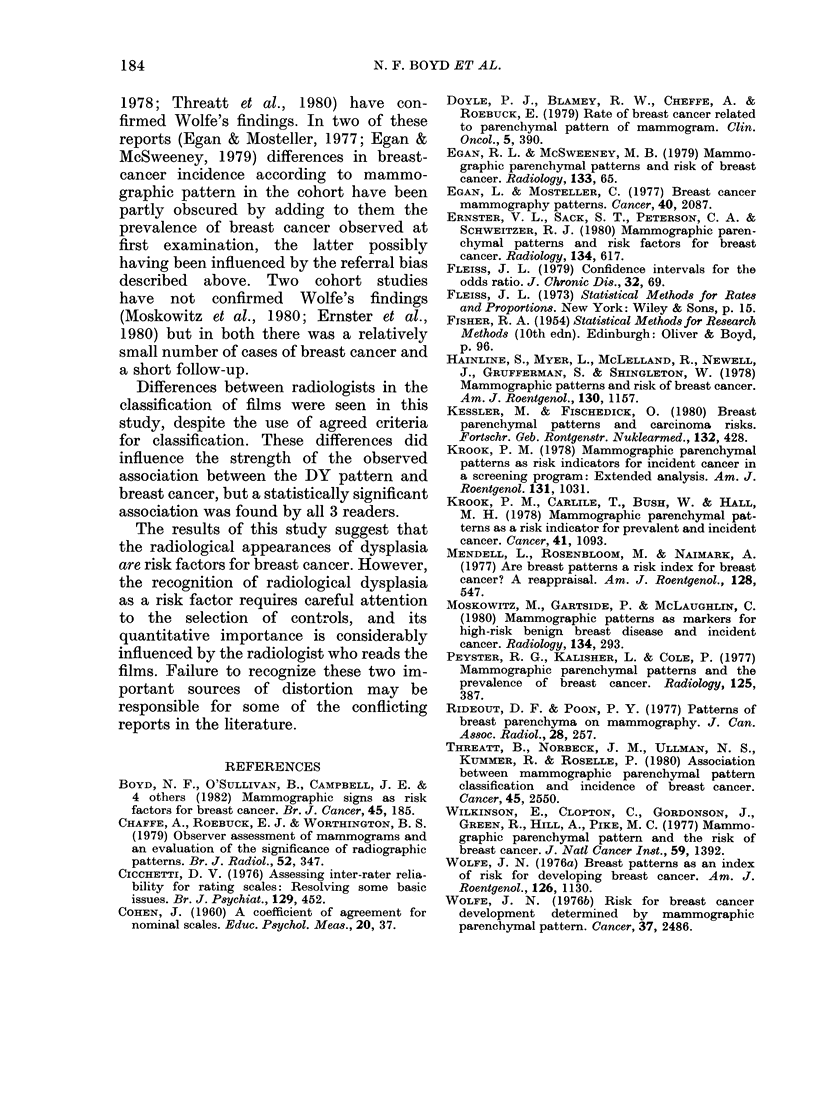# Bias and the association of mammographic parenchymal patterns with breast cancer.

**DOI:** 10.1038/bjc.1982.31

**Published:** 1982-02

**Authors:** N. F. Boyd, B. O'Sullivan, J. E. Campbell, E. Fishell, I. Simor, G. Cooke, T. Germanson

## Abstract

We have carried out a case-control study to evaluate the association between Wolfe's mammographic patterns and the risk of breast cancer, and to examine the influence of control selection and the radiologist who read the films upon the results obtained. Mammograms of the non-cancerous breast of 183 women with unilateral breast cancer were compared with mammograms from two age-matched control groups: a group of asymptomatic women attending a screening centre, and a group of symptomatic women referred for the diagnostic evaluation of suspected breast disease. Films were arranged in random sequence and independently classified by 3 radiologists. A strong and statistically significant association was found between mammographic dysplasia and breast cancer when controls from the screening centre were compared to cases, but not when cases were compared to women referred for the diagnostic evaluation of breast disease. This result appears to arise in part because of an association between symptoms of benign breast disease and mammographic dysplasia, and suggests that some previous negative studies of the association of mammographic patterns with breast cancer may have arisen from the inclusion of symptomatic subjects as controls.


Br. J. Cancer (1982) 45, 179

### BIAS AND THE ASSOCIATION OF MAMMOGRAPHIC PARENCHYMAL PATTERNS WITH BREAST CANCER

N. F. BOYD*, B. O'SULLIVAN*, J. E. CAMPBELL*, E. FISHELL‡, I. SIMOR§, G. COOKE¶ AND T. GERMANSON†

*From the Departments of *Medicine and †Biostatistics, Princess Margaret Hospital, and Departments of Radiology, ‡Women's College Hospital, §Mount Sinai Hospital, and ¶St Michael's Hospital, Toronto, Ontario, Canada*

Received 27 April 1981 Accepted 13 October 1981

**Summary.—We have carried out a case–control study to evaluate the association between Wolfe's mammographic patterns and the risk of breast cancer, and to examine the influence of control selection and the radiologist who read the films upon the results obtained.**

**Mammograms of the non-cancerous breast of 183 women with unilateral breast cancer were compared with mammograms from two age-matched control groups: a group of asymptomatic women attending a screening centre, and a group of symptomatic women referred for the diagnostic evaluation of suspected breast disease. Films were arranged in random sequence and independently classified by 3 radiologists.**

**A strong and statistically significant association was found between mammographic dysplasia and breast cancer when controls from the screening centre were compared to cases, but not when cases were compared to women referred for the diagnostic evaluation of breast disease. This result appears to arise in part because of an association between symptoms of benign breast disease and mammographic dysplasia, and suggests that some previous negative studies of the association of mammographic patterns with breast cancer may have arisen from the inclusion of symptomatic subjects as controls.**

WOLFE HAS DESCRIBED a method of classifying the mammographic appearances of ductal prominence and dysplasia that is reported to identify individuals at different risks for the development of breast cancer (Wolfe, 1976*a,b*). However, not all subsequent studies of these mammographic patterns by other investigators have confirmed this finding. A review of these studies has revealed several differences in research design and methodology that might explain the conflicting results. Some studies report the incidence of breast cancer in a cohort of women classified according to their initial mammographic characteristics (Egan & Mosteller, 1977; Egan & McSweeney, 1979; Krook *et al.*, 1978; Krook, 1978; Threatt *et al.*, 1980; Moskowitz *et al.*, 1980; Ernster *et al.*, 1980). In others, the prevalence of breast cancer detected at the first examination within each category of mammographic pattern is described (Egan & Mosteller, 1977; Krook, 1978; Kessler & Fischedick, 1980). Still others have used a case–control design, not always with a formally constituted control group, in which the prevalence of each mammographic pattern in a group of women with diagnosed breast cancer is compared to that of

Correspondence to: Dr N. F. Boyd, Department of Medicine, Princess Margaret Hosptial, 500 Sherbourne Street, Toronto, Ontario, Canada.

controls (Mendell *et al.*, 1977; Rideout & Poon, 1977; Hainline *et al.*, 1978; Wilkinson *et al.*, 1977; Doyle *et al.*, 1979; Chaffe *et al.*, 1979).

There are further differences within each of these designs. Subjects have sometimes been drawn from diagnostic centres and sometimes from screening centres, and these groups may differ with respect to both their risk for breast cancer and other attributes. The classification of mammograms into the groups described by Wolfe has generally been performed by only 1–2 radiologists at a single institution, which, in view of the subjective nature of the classification, raises the possibility that other readers classifying the same films might obtain different results. Finally, the classification of mammograms has not always been made without knowledge of which films came from women with breast cancer, and such knowledge might influence the reader's classification of the films.

We carried out this case–control study to determine whether an association between breast cancer and the mammographic patterns described by Wolfe could be identified when the radiologist classifying the films was unaware which came from cases and which from controls. Further, we wished to examine the possible influence upon the results of some methodological issues, particularly the selection of controls, as well as the reproducibility of the result between different radiologists independently classifying the same films. This report is concerned primarily with these methodological questions. The influence of other factors, including age and menopausal status, and a more detailed examination of mammographic signs as risk factors for breast cancer, are addressed in an accompanying paper (Boyd *et al.*, 1982).

### MATERIALS AND METHODS

We selected 2 groups of controls, one from a screening centre and the other from a diagnostic referral service, and a group of histologically verified breast-cancer cases. Details of the selection of controls from a screening centre (screened controls) and of patients with breast cancer, and the criteria used to classify films, are given in the accompanying paper (Boyd *et al.*, 1982) and will not be repeated here.

Controls from a diagnostic referral service (diagnostic controls) were selected from ~ 2400 patients referred to the breast diagnostic service of Women's College Hospital during the years 1977–78 who were considered to be free of breast cancer after evaluation. Patients were generally referred because of suspected breast disease, and most had symptoms. Many also had abnormalities on physical examination. Patients were randomly selected from the total group of referred patients, and retained in the diagnostic control group if they had had a mammogram, were aged 40–65 years, and could be age-matched to a case. If any of these criteria could not be met the subject was rejected and another random selection made. The data systematically collected at the time of the patients' attendance at the breast diagnostic service included information about breast symptoms.

Both control groups and the case group had had mammograms in the Department of Radiology at Women's College Hospital. Because the mammogram from the cancerous breast of the cases was expected to show radiological signs of malignancy, which would have alerted the radiologist to their identity, we chose the film from the non-cancerous breast. The mammograms from these 3 groups were then arranged in random sequence and the pattern of the parenchyma classified independently by 3 of us at different institutions, without knowledge of which films were from cases and which from controls. Five hundred and thirty-two of the 549 mammograms (97%) used in this study were xeromammograms.

*Statistical procedures.*—The association between breast cancer and the parenchymal patterns was assessed using the odds ratio. The statistical significance of the observed association was tested by $\chi^2$, calculated as described by Fleiss (1973). The $P$ values cited were calculated by Fisher's exact test (Fisher, 1954). Ninety-five per cent confidence intervals were calculated for the odds ratios, using the iterative technique of Cornfield (Fleiss, 1979). Agreement between radiologists on the classification of films was assessed using the weighted Kappa statistic (Cohen, 1960; Cicchetti, 1976).

### RESULTS

To examine the association between mammographic patterns and breast cancer we compared the prevalence of the patterns among the breast cases with the screened control group and the diagnostic control group. Matched and unmatched analyses were made, with very similar results; only the results of unmatched comparisons will be shown.

### *Comparison of screened control group and breast cancer cases*

Table I shows the prevalence of each of the mammographic parenchymal patterns in the screened control group and the case group, according to each of the 3 radiologists who classified the films. Each radiologist identified the dysplasia (DY) pattern more often in cases than controls. Radiologist A classified 78/183 cases (43%) as having the DY pattern, compared to 44/183 controls (24%), and Radiologist C classified the films in a similar way. Radiologist B classified more films from both the cases (105/183, 57%) and controls (80/183, 44%) as having the DY pattern, but still identified the DY pattern more often among the cases. The odds ratio for the association between the DY pattern and breast cancer exceeded unity with statistical significance for each reader, and the 95%-confidence intervals for this odds ratio were A: 1·82–5·98; B: 1·07–3·32 and C: 1·97–6·85.

Each radiologist classified a similar number of cases and controls as having the $P_2$ parenchymal pattern. Radiologists A and C found an association between the $P_2$ pattern and breast cancer that was of border line significance, and a weak non-significant association between the $P_1$ pattern and breast cancer.

### *Comparison of diagnostic control group and breast-cancer cases*

Table II shows the prevalence of each of the mammographic parenchymal patterns in the diagnostic control group and the case group according to the 3 radiologists. Each reader classified the mammograms from controls and cases in a similar way, and there were no significant associations between the case group and any of the mammographic patterns.

### *Evidence for referral bias affecting the diagnostic controls*

These results show that the demonstration of an association between the DY mammographic pattern and breast cancer in this case-control study is critically dependent on the selection of the controls.

The true prevalence of the DY mammo-

TABLE I.—*Comparison of screened controls and cases according to mammographic pattern and radiologist*

| Radiologist | Pattern | Controls | Cases | Odds* ratio | $\chi^2$ | $P$ |
|---|---|---|---|---|---|---|
| A | N | 54 | 29 | 1·0 | — | — |
| | $P_1$ | 33 | 24 | 1·35 | 0·46 | 0·49 |
| | $P_2$ | 52 | 52 | 1·86 | 3·67 | 0·05 |
| | DY | 44 | 78 | 3·30 | 15·50 | 0·00007 |
| B | N | 46 | 32 | 1·0 | — | — |
| | $P_1$ | 26 | 15 | 0·83 | 0·07 | 0·79 |
| | $P_2$ | 31 | 31 | 1·44 | 0·79 | 0·37 |
| | DY | 80 | 105 | 1·88 | 4·82 | 0·03 |
| C | N | 48 | 24 | 1·0 | — | — |
| | $P_1$ | 64 | 50 | 1·56 | 1·62 | 0·20 |
| | $P_2$ | 28 | 30 | 2·14 | 3·75 | 0·05 |
| | DY | 43 | 79 | 3·67 | 16·71 | 0·00004 |

* Odds ratios, $\chi^2$ and $P$ are calculated for each pattern with reference to the N pattern.

TABLE II.—*Comparison of diagnostic controls and cases according to mammographic pattern and radiologist*

| Radiologist | Pattern | Controls | Cases | Odds* ratio | $\chi^2$ | $P$ |
|---|---|---|---|---|---|---|
| A | N | 24 | 29 | 1·0 | — | — |
| | $P_1$ | 25 | 24 | 0·79 | 0·14 | 0·70 |
| | $P_2$ | 63 | 52 | 0·68 | 0·96 | 0·33 |
| | DY | 71 | 78 | 0·91 | 0·02 | 0·89 |
| B | N | 27 | 32 | 1·0 | — | — |
| | $P_1$ | 15 | 15 | 0·84 | 0·02 | 0·44 |
| | $P_2$ | 31 | 31 | 0·84 | 0·08 | 0·77 |
| | DY | 110 | 105 | 0·80 | 0·34 | 0·55 |
| C | N | 26 | 24 | 1·0 | — | — |
| | $P_1$ | 45 | 50 | 1·20 | 0·13 | 0·72 |
| | $P_2$ | 35 | 30 | 0·93 | 0·00 | 0·99 |
| | DY | 77 | 79 | 1·11 | 0·03 | 0·87 |

* Odds ratios, $\chi^2$ and $P$ are calculated for each pattern with reference to the N pattern.

graphic pattern in the community is most closely approximated in this study by the group of screened controls who were volunteers from the general population. We have examined possible explanations for the high prevalence of the DY pattern in the diagnostic controls. Many patients in the diagnostic control group had symptoms of breast disease, such as a breast lump (128; 70%), breast pain (73; 40%), and nipple discharge (12; 7%), which are commonly associated with the clinical entity of mammary dysplasia, and are frequently the stimulus for diagnostic referral. These symptoms were less common in the screened control group: 7 patients (4%) reported noting a breast lump in the previous 6 months, 35 (19%) reported breast pain, and 3 (2%), reported a nipple discharge.

To determine whether there was an association between the DY pattern and symptoms of breast disease that might explain the selective referral of patients with the DY pattern, we analysed the relationship between DY pattern and these symptoms. Each symptom was found more often in patients with the DY pattern, and the strongest association was with breast lump. We then compared the group of breast-cancer cases with each control group after stratification according to the presence or absence of a breast lump.

Table III shows the distribution of the DY pattern among the diagnostic controls and breast-cancer cases after stratification according to the presence or absence of a breast lump. (Cases "without a breast lump" were those detected by routine examination while asymptomatic, or who presented with other symptoms, or who were detected by mammography alone.) The odds ratio was close to unity when cases and controls with a breast lump were compared, but rose to 2·97 (approaching conventional levels of statistical significance at a 2-sided level) when cases and controls without a breast lump were compared. A similar comparison of screened controls with breast-cancer cases showed that a statistically significant association between DY pattern and breast cancer persisted in those without the symptom of a breast lump, but was lost when those with a breast lump were compared.

TABLE III.—*Distribution of pattern in diagnostic controls and cases according to symptom of breast lump*

| | Breast lump | | | |
|---|---|---|---|---|
| | Present | | Absent | |
| Pattern | Controls | Cases | Controls | Cases |
| Non-DY | 73 | 95 | 38 | 8 |
| DY | 55 | 66 | 16 | 10 |
| Total | 128 | 161 | 54 | 18 |
| Odds ratio | 0·92 | | 2·97 | |
| $\chi^2$ | 0·75 | | 2·89 | |
| $P$ | 0·39 | | 0·08 | |

It thus seems likely that the referral bias observed in the diagnostic control group arose, at least in part, because of a relationship between the DY mammographic pattern and symptoms of breast disease.

*Observer variation in the classification of mammographic patterns*

Table IV shows agreement between Radiologists A and B on the classification of the total set of 549 films. These two readers agreed exactly on the classification of 366 of these films (67%). If credit for some categories of disagreement is taken into account, using weights proposed by Cicchetti for continuous-ordinal classifications, the proportion of agreement rises to 84%. (In using these weights, cells of perfect agreement are multiplied by 1, those one category apart by 0·66, and those two categories apart by 0·33; classifications 3 categories apart are given a weight of 0. The sum of the products of cell number and weight is divided by the total number of films to give the proportion of weighted agreement.) Further, taking into account the amount of agreement expected by chance using the weighted Kappa statistic, Kappa was 0·62 for this pair of readers indicating agreement significantly greater than expected by chance. Values of weighted Kappa for the other two pairs of readers were 0·60 for A and C, and 0·61 for B and C.

TABLE IV.—*Observer variation in the classification of mammographic patterns: Radiologists A and B*

| Radiologist A | Radiologist B | | | | |
|---|---|---|---|---|---|
| | N | $P_1$ | $P_2$ | DY | Total |
| N | 80 | 10 | 5 | 12 | 107 |
| $P_1$ | 12 | 34 | 9 | 27 | 82 |
| $P_2$ | 0 | 11 | 76 | 80 | 167 |
| DY | 13 | 1 | 3 | 176 | 193 |
| Total | 105 | 56 | 93 | 295 | 549 |

### DISCUSSION

These results show a strong association between the DY mammographic pattern of Wolfe's classification and breast cancer, when the mammographic patterns of breast-cancer cases are compared to those of largely asymptomatic controls from a screening centre. However, this association is not seen when the comparison group consists of symptomatic women attending a diagnostic referral service. This difference in results appears to arise, at least in part, from an association between the DY pattern and symptoms of breast disease in the absence of breast cancer, which gives rise to the selective referral of women with the DY pattern.

As a consequence of this referral bias, any comparison of mammographic patterns of patients with breast cancer with a control group in whom symptoms of breast disease are common is likely to underestimate the risk associated with the DY pattern. Similarly, any analysis based on the distribution of prevalent cancers in a patient population referred for examination because of breast symptoms is also liable to underestimate the risk, because a large proportion of the population, with or without breast cancer, can be expected to have the DY pattern. Several of the published reports that have failed to confirm the DY pattern as a risk factor for breast cancer have involved patients referred because of suspected breast disease (Peyster *et al.*, 1977; Kessler & Fishedick, 1980; Rideout & Poon, 1977; Doyle *et al.*, 1979). Other case–control studies from screening centres where a low prevalence of breast symptoms would be expected in the control group, have generally confirmed the DY pattern as a risk factor for breast cancer (Hainline *et al.*, 1978; Wilkinson *et al.*, 1977; Chaffe *et al.*, 1979).

Cohort studies are less susceptible to the form of referral bias identified in this study. Wolfe's two original reports of the association of parenchymal pattern with breast cancer were based upon cohorts and 5 subsequent studies of this type (Egan & Mosteller, 1977; Egan & McSweeney, 1979; Krook *et al.*, 1978; Krook,

1978; Threatt *et al.*, 1980) have confirmed Wolfe's findings. In two of these reports (Egan & Mosteller, 1977; Egan & McSweeney, 1979) differences in breast-cancer incidence according to mammographic pattern in the cohort have been partly obscured by adding to them the prevalence of breast cancer observed at first examination, the latter possibly having been influenced by the referral bias described above. Two cohort studies have not confirmed Wolfe's findings (Moskowitz *et al.*, 1980; Ernster *et al.*, 1980) but in both there was a relatively small number of cases of breast cancer and a short follow-up.

Differences between radiologists in the classification of films were seen in this study, despite the use of agreed criteria for classification. These differences did influence the strength of the observed association between the DY pattern and breast cancer, but a statistically significant association was found by all 3 readers.

The results of this study suggest that the radiological appearances of dysplasia *are* risk factors for breast cancer. However, the recognition of radiological dysplasia as a risk factor requires careful attention to the selection of controls, and its quantitative importance is considerably influenced by the radiologist who reads the films. Failure to recognize these two important sources of distortion may be responsible for some of the conflicting reports in the literature.

### REFERENCES

Boyd, N. F., O'Sullivan, B., Campbell, J. E. & 4 others (1982) Mammographic signs as risk factors for breast cancer. *Br. J. Cancer*, **45**, 185.

Chaffe, A., Roebuck, E. J. & Worthington, B. S. (1979) Observer assessment of mammograms and an evaluation of the significance of radiographic patterns. *Br. J. Radiol.*, **52**, 347.

Cicchetti, D. V. (1976) Assessing inter-rater reliability for rating scales: Resolving some basic issues. *Br. J. Psychiat.*, **129**, 452.

Cohen, J. (1960) A coefficient of agreement for nominal scales. *Educ. Psychol. Meas.*, **20**, 37.

Doyle, P. J., Blamey, R. W., Cheffe, A. & Roebuck, E. (1979) Rate of breast cancer related to parenchymal pattern of mammogram. *Clin. Oncol.*, **5**, 390.

Egan, R. L. & McSweeney, M. B. (1979) Mammographic parenchymal patterns and risk of breast cancer. *Radiology*, **133**, 65.

Egan, L. & Mosteller, C. (1977) Breast cancer mammography patterns. *Cancer*, **40**, 2087.

Ernster, V. L., Sack, S. T., Peterson, C. A. & Schweitzer, R. J. (1980) Mammographic parenchymal patterns and risk factors for breast cancer. *Radiology*, **134**, 617.

Fleiss, J. L. (1979) Confidence intervals for the odds ratio. *J. Chronic Dis.*, **32**, 69.

Fleiss, J. L. (1973) *Statistical Methods for Rates and Proportions*. New York: Wiley & Sons, p. 15.

Fisher, R. A. (1954) *Statistical Methods for Research Methods* (10th edn). Edinburgh: Oliver & Boyd, p. 96.

Hainline, S., Myer, L., McLelland, R., Newell, J., Grufferman, S. & Shingleton, W. (1978) Mammographic patterns and risk of breast cancer. *Am. J. Roentgenol.*, **130**, 1157.

Kessler, M. & Fischedick, O. (1980) Breast parenchymal patterns and carcinoma risks. *Fortschr. Geb. Rontgenstr. Nuklearmed.*, **132**, 428.

Krook, P. M. (1978) Mammographic parenchymal patterns as risk indicators for incident cancer in a screening program: Extended analysis. *Am. J. Roentgenol.* **131**, 1031.

Krook, P. M., Carlile, T., Bush, W. & Hall, M. H. (1978) Mammographic parenchymal patterns as a risk indicator for prevalent and incident cancer. *Cancer*, **41**, 1093.

Mendell, L., Rosenbloom, M. & Naimark, A. (1977) Are breast patterns a risk index for breast cancer? A reappraisal. *Am. J. Roentgenol.*, **128**, 547.

Moskowitz, M., Gartside, P. & McLaughlin, C. (1980) Mammographic patterns as markers for high-risk benign breast disease and incident cancer. *Radiology*, **134**, 293.

Peyster, R. G., Kalisher, L. & Cole, P. (1977) Mammographic parenchymal patterns and the prevalence of breast cancer. *Radiology*, **125**, 387.

Rideout, D. F. & Poon, P. Y. (1977) Patterns of breast parenchyma on mammography. *J. Can. Assoc. Radiol.*, **28**, 257.

Threatt, B., Norbeck, J. M., Ullman, N. S., Kummer, R. & Roselle, P. (1980) Association between mammographic parenchymal pattern classification and incidence of breast cancer. *Cancer*, **45**, 2550.

Wilkinson, E., Clopton, C., Gordonson, J., Green, R., Hill, A., Pike, M. C. (1977) Mammographic parenchymal pattern and the risk of breast cancer. *J. Natl Cancer Inst.*, **59**, 1392.

Wolfe, J. N. (1976*a*) Breast patterns as an index of risk for developing breast cancer. *Am. J. Roentgenol.*, **126**, 1130.

Wolfe, J. N. (1976*b*) Risk for breast cancer development determined by mammographic parenchymal pattern. *Cancer*, **37**, 2486.